# Interpretable machine learning models for detecting peripheral neuropathy and lower extremity arterial disease in diabetics: an analysis of critical shared and unique risk factors

**DOI:** 10.1186/s12911-024-02595-z

**Published:** 2024-07-22

**Authors:** Ya Wu, Danmeng Dong, Lijie Zhu, Zihong Luo, Yang Liu, Xiaoyun Xie

**Affiliations:** 1 Department of Endocrinology and Metabolism, Shanghai Tenth People’s Hospital, Tongji University School of Medicine, Shanghai, China; 2 School of Medicine, Anhui University of Science and Technology, Huainan, China; 3 School of Advanced Technology, Xi’an Jiaotong-Liverpool University, Suzhou, China; 4 Department of Geriatrics, Tongji Hospital, Tongji University School of Medicine, Shanghai, China

**Keywords:** Diabetic peripheral neuropathy (DPN), Lower extremity arterial disease (LEAD), Machine learning (ML), Risk factor

## Abstract

**Background:**

Diabetic peripheral neuropathy (DPN) and lower extremity arterial disease (LEAD) are significant contributors to diabetic foot ulcers (DFUs), which severely affect patients’ quality of life. This study aimed to develop machine learning (ML) predictive models for DPN and LEAD and to identify both shared and distinct risk factors.

**Methods:**

This retrospective study included 479 diabetic inpatients, of whom 215 were diagnosed with DPN and 69 with LEAD. Clinical data and laboratory results were collected for each patient. Feature selection was performed using three methods: mutual information (MI), random forest recursive feature elimination (RF-RFE), and the Boruta algorithm to identify the most important features. Predictive models were developed using logistic regression (LR), random forest (RF), and eXtreme Gradient Boosting (XGBoost), with particle swarm optimization (PSO) used to optimize their hyperparameters. The SHapley Additive exPlanation (SHAP) method was applied to determine the importance of risk factors in the top-performing models.

**Results:**

For diagnosing DPN, the XGBoost model was most effective, achieving a recall of 83.7%, specificity of 86.8%, accuracy of 85.4%, and an F1 score of 83.7%. On the other hand, the RF model excelled in diagnosing LEAD, with a recall of 85.7%, specificity of 92.9%, accuracy of 91.9%, and an F1 score of 82.8%. SHAP analysis revealed top five critical risk factors shared by DPN and LEAD, including increased urinary albumin-to-creatinine ratio (UACR), glycosylated hemoglobin (HbA1c), serum creatinine (Scr), older age, and carotid stenosis. Additionally, distinct risk factors were pinpointed: decreased serum albumin and lower lymphocyte count were linked to DPN, while elevated neutrophil-to-lymphocyte ratio (NLR) and higher D-dimer levels were associated with LEAD.

**Conclusions:**

This study demonstrated the effectiveness of ML models in predicting DPN and LEAD in diabetic patients and identified significant risk factors. Focusing on shared risk factors may greatly reduce the prevalence of both conditions, thereby mitigating the risk of developing DFUs.

**Supplementary Information:**

The online version contains supplementary material available at 10.1186/s12911-024-02595-z.

## Introduction

Diabetes has become a growing global health concern, affecting around 451 million people in 2017, with projections to rise to 693 million by 2045 [1]. In China, the prevalence of diabetes has surged from less than 1% in the 1980s to approximately 10.9% in 2013 and 12.4% in 2018, making it the country with the world’s largest diabetic population [2]. Diabetic peripheral neuropathy (DPN) and lower extremity arterial disease (LEAD) are prevalent complications of diabetes, with occurrence rates of about 50% and 3-20%, respectively [3–6]. Both complications serve as extrinsic risk factors for diabetic foot ulcers (DFUs) [7], leading to higher rates of amputation, increased mortality, and substantial economic burdens for patients with diabetes [8]. Unfortunately, patients with DPN or LEAD may be asymptomatic in their early stages, and many patients already have these complications at the time of initial diagnosis [8, 9]. Therefore, early identification and management of DPN and LEAD are crucial in preventing DFUs among diabetic patients.

At present, the diagnosis of DPN and LEAD mainly relies on physical examination of the peripheral nervous system, electromyography (EMG), ankle–brachial index, lower limb vascular ultrasound, etc. [5, 10]. However, these methods require well-trained endocrinologists and specialized diagnostic equipment, which are often scarce in underdeveloped regions. To address this challenge, researchers are actively exploring the development of practical, accessible, and cost-effective clinical diagnosis models for DPN and LEAD based on clinical features and routinely measured lab parameters. Recent studies have demonstrated that machine learning (ML) models, by utilizing medical history, physical examinations, and basic lab tests, could effectively predict DPN and LEAD [4, 11]. Moreover, ML algorithms achieved high accuracy in identifying DPN through the analysis of immune biomarkers or microcirculatory parameters [12, 13]. Additionally, a model based on support vector machine (SVM) has been shown to accurately predict DPN severity in about 76% of cases, utilizing general patient information and responses from a neuropathy disability score questionnaire [14]. Despite these advances, most studies have concentrated on developing one model for either DPN or LEAD, without considering common risk factors for both conditions in a single study. Given the high prevalence of DPN and LEAD in developing countries and their potential to lead to DFUs, which can significantly increase mortality rates [15, 16], it is crucial to identify and target shared risk factors for both conditions. This strategy could facilitate early and concurrent interventions, potentially diminishing the prevalence and severity of these diseases.

In this study, we employed logistic regression (LR), random forest (RF), and eXtreme Gradient Boosting (XGBoost) to develop diagnostic models for both DPN and LEAD among diabetic individuals, utilizing demographic, clinical, and laboratory information. This research spanned the interdisciplinary fields of medicine, biostatistics, and ML. To identify shared and unique risk factors for DPN and LEAD, we used the SHapley Additive exPlanation (SHAP) method to prioritize risk factors within the most effective models for each condition.

## Contributions of this work

The major contributions of this study are outlined as follows:

1. We constructed ML models for DPN and LEAD detection based on accessible demographic, clinical, and laboratory data, minimizing the need for specialized tests and advanced medical facilities. This approach is especially beneficial for areas with limited healthcare resources.
2. We utilized three feature selection methods—mutual information (MI), random forest recursive feature elimination (RF-RFE), and the Boruta algorithm—to identify the most significant features. This strategy effectively reduces overfitting and enhances the robustness of our models.
3. To optimize the performance of each ML model, we applied particle swarm optimization (PSO) to fine-tune hyperparameters.
4. The SHAP method was applied to elucidate the contribution of each feature to the risk of developing DPN and LEAD in the best-performing models. This analysis identified both shared and distinct risk factors for DPN and LEAD, deepening our insight into their pathophysiological foundations. Concentrating on shared risk factors may significantly reduce the prevalence of these conditions and subsequently the risk of DFUs.

## Methods

### Study design and participants

This is a cross-sectional study conducted at Tongji Hospital from January 2022 to March 2023. We collected clinical characteristics and laboratory data of 712 diabetic inpatients who underwent EMG and lower limb vascular ultrasound examinations. Patients were excluded from the study if they had diabetic ketoacidosis (DKA), hyperosmotic hyperglycemia syndrome (HHS), autoimmune diseases, infectious diseases, malignant tumors, or if more than 30% of their data was missing. Ultimately, 479 diabetic patients were enrolled in this study. This research was performed in compliance with the Code of Ethics of the World Medical Association (Declaration of Helsinki) and received approval from the Institutional Ethics Committee (K-2023-022).

### Diagnostic criteria

All diabetic patients enrolled in our study underwent screening for DPN and LEAD during hospitalization. The diagnosis of diabetic complications was made by two qualified endocrinologists based on the locally recognized criteria [17]. A Dantec® Keypoint® G4 EMG/NCS/EP Workstation with 8-Channel Amplifier (Dantec Medical A/S, Denmark) was used to test the motor conduction velocity of bilateral ulnar, median, and common peroneal nerves, as well as the sensory conduction velocity of bilateral radial, median, and superficial fibular nerves. The diagnosis of DPN relied on typical symptoms, neurological examination, EMG, and exclusion of other causes of peripheral neuropathy. In addition, Philips IU22 Doppler ultrasonic color imaging system (Philips, USA) equipped with a 3-D array probe (5-12 MHz) was applied to examine the bilateral common femoral arteries, superficial femoral artery, popliteal artery, and dorsal artery. The diagnosis of LEAD was based on arterial lumen stenosis, severe blood flow filling deficiency, or arterial occlusion.

### Data collection

Demographic information was collected on admission, including age, gender, body mass index (BMI), diabetes duration, diabetes type, smoking history, systolic blood pressure (SBP) and diastolic blood pressure (DBP). BMI was calculated by dividing an individual’s weight in kilograms by the square of their height in meters. SBP and DBP were measured three times while the person was at rest, and then the average of the readings was recorded. Clinical routine examinations were performed, and laboratory parameters were obtained from fasting blood and spot urine samples collected the next morning after admission. The routine laboratory tests included hematology (neutrophil, lymphocyte, and platelet counts, neutrophil-to-lymphocyte ratio (NLR)), liver and kidney function tests (alanine aminotransferase (ALT), aspartate aminotransferase (AST), serum albumin, total bilirubin (TBiL), direct bilirubin (DBiL), serum urea nitrogen (SUN), serum uric acid (SUA), serum creatinine (Scr)), glucose metabolism (fasting blood glucose (FBG), glycosylated hemoglobin (HbA1c)), lipid profiles (total cholesterol (TC), high-density lipoprotein cholesterol (HDL-C), low-density lipoprotein cholesterol (LDL-C), and triglycerides (TG)), islet function (fasting insulin and fasting C-peptide), C-reactive protein (CRP), D-dimer, 25-hydroxy vitamin D3 (25-OH VitD), ferritin, neuron specific enolase (NSE), urinary microalbumin and creatinine, and the urinary albumin-to-creatinine ratio (UACR). We calculated the estimated glomerular filtration rate (eGFR) using a formula provided in a previous study [18]. In addition, we collected the results of carotid artery ultrasound. Bilateral common carotid artery, internal carotid artery, and external carotid artery were examined using a Philips IU22 Doppler ultrasonic color imaging system (Philips, USA) equipped with a 3-D array probe (7-12 MHz). The dataset, encompassing all features and their respective values, was detailed in Supplementary Table S1.

### Missing data

Comprehensive demographic information was available for all participants, as each patient underwent the hospitalization process. Missing data for laboratory parameters were below 30%. We addressed these missing values using the most recent available measurements. Any remaining missing values were imputed using the median.

### Data balancing

In constructing models for LEAD, we encountered a class imbalance issue due to the low proportion of patients with LEAD in the overall population. To address this problem, we used the imbalanced-learn package in Python to employ a random undersampling technique, achieving a 1:3 ratio between the LEAD and non-LEAD groups [19]. This approach helped us to maintain a more balanced ratio and decrease the number of non-LEAD cases to three times the number of LEAD cases, thus mitigating the imbalance and enhancing the reliability of our models.

### Feature selection strategy

The feature selection process was conducted using three distinct methods: MI, RF-RFE, and the Boruta algorithm. MI quantifies the dependency between variables by capturing all types of relationships, both linear and nonlinear. For feature selection, MI assesses the dependency of each feature on the target label to identify the most informative features for prediction [20]. RF-RFE utilizes a RF to iteratively build models, systematically removing the least important features in each round. This method emphasizes features that significantly affect model performance [21]. The Boruta algorithm employs a RF classifier to evaluate features against their randomized “shadow” versions, ensuring only essential features are retained for accurate model predictions [22].

For both MI and RF-RFE, the top 15 features were identified independently. The Boruta algorithm categorized features as confirmed, tentative, or rejected, selecting features that were either confirmed or tentative. Only features chosen by at least two of these three methods were used to develop ML models. This approach reduces redundancy and enhances the predictive accuracy of the ML models.

### ML model construction and interpretation

**Fig. 1:**
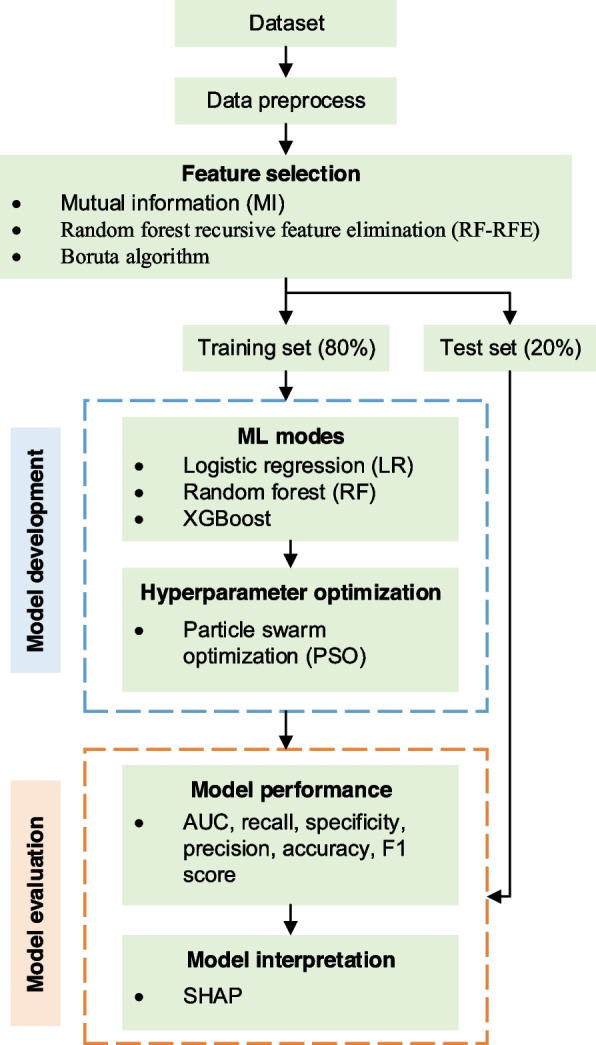
ML model development and evaluation process

The model was constructed and interpreted using Python (version 3.9.6, Python Software Foundation, USA). The workflow for constructing and interpreting ML models is illustrated in Fig. 1. First, the dataset was randomly divided into two subsets: 80% designated for training the model and the remaining 20% reserved for testing. Then, three distinct ML models—LR, RF, and XGBoost—were developed to predict DPN and LEAD based on selected features. To optimize these models and select the most suitable hyperparameters, we employed PSO. PSO is a computational method that mimics a swarm of particles navigating through the parameter space to find optimal solutions. In ML, PSO enhances model parameterization by representing each particle as a potential solution that is continuously refined through both individual and collective experiences within the swarm. This strategy efficiently identifies the best parameter combinations, significantly improving model performance [23]. The set and optimal value of hyperparameters were displayed in Supplementary Table S2.

The effectiveness of each model was evaluated using various metrics, including recall, specificity, precision, accuracy, and the F1 score. We also calculated the area under the receiver operating curve (AUC) for the test sets to assess the performance of each model. Furthermore, the SHAP method was employed to interpret the contribution of each predictor within the optimal models. Through SHAP analysis, we gained a detailed understanding of how each feature influences the model’s output, providing a comprehensive insight into the model’s decision-making process [24].

### Statistical analysis

The statistical analyses were performed using SPSS (version 27.0, IBM, USA). For data adhering to a normal distribution, values were depicted as mean ± standard deviation. Differences among these values were examined using the independent Student’s *t*-test. Conversely, for data not following a normal distribution, variables were presented as medians (interquartile range, IQR), and the Mann-Whitney U test was employed to evaluate disparities in their distributions. Categorical data were represented as n (%) and analyzed for distribution differences via the Chi-square (χ^2^) test or Fisher’s exact test, as appropriate. A *p*-value < 0.05 was considered statistically significant.

## Results

### Clinical features of patients

**Fig. 2:**
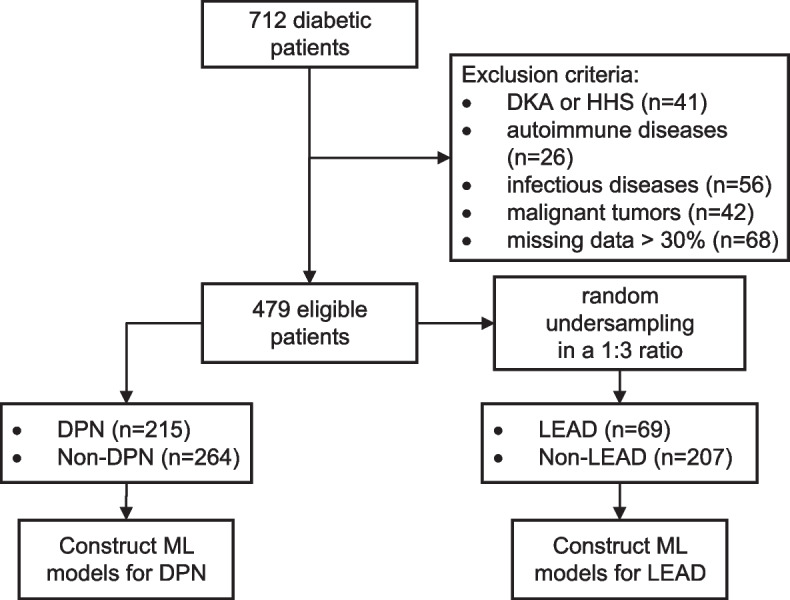
Flowchart of patient enrollment. DPN, diabetic peripheral neuropathy. LEAD, lower extremity arterial disease. DKA, diabetic ketoacidosis. HHS, hyperosmotic hyperglycemia syndrome. ML, machine learning

In this study, we initially enrolled 712 diabetic inpatients. After applying exclusion criteria, 479 patients qualified for inclusion. Among them, 215 were diagnosed with DPN, and 69 with LEAD. The median age of participants was 50 years (IQR: 48-56), and the male-to-female ratio was 0.58. All these cases were utilized to develop models for diagnosing DPN. To correct for the imbalance in sample sizes, a one-to-three random undersampling strategy was employed for LEAD cases versus non-LEAD controls, resulting in 69 LEAD cases and 207 non-LEAD cases being selected to construct LEAD prediction models (Fig. 2). According to univariate analysis, out of the 38 features, 24 exhibited significant discrepancies between patients with and without DPN, whereas 17 features displayed differences between those with and without LEAD (Tables 1 and 2). Patients with DPN or LEAD were found to be older compared to those without these complications. Men were more likely to develop DPN or LEAD than women. Moreover, patients with DPN and LEAD exhibited increased levels of HbA1c, SUN, Scr, FBG, D-dimer, NLR, urinary microalbumin, and UACR compared to those without these complications. In contrast, the levels of serum albumin, eGFR, TC, and LDL were found to be lower in patients with DPN and LEAD. Additionally, a positive association was observed between the presence of carotid stenosis and the occurrence of DPN and LEAD.

**Table 1:** Clinical features in diabetic patients with or without DPN

Clinical features	Non-DPN (*n* = 264)	DPN (*n* = 215)	*P* value
Demographic profiles			
Age (years)	64.00 (56.25, 68.00)	66.00 (61.00, 72.00)	< 0.001
Sex, n (%)			< 0.001
Male	120 (45.5)	146 (67.9)	
Female	144 (54.5)	69 (32.1)	
BMI (kg/m^2^)	24.48 (22.64, 27.17)	23.88 (21.83, 26.06)	0.006
Diabetes duration (year)	10.00 (4.63, 16.00)	15.00 (8.00, 20.00)	< 0.001
Type			0.338
T1DM	4 (1.5)	7 (3.3)	
T2DM	260 (98.5)	208 (96.7)	
Smoking history, n (%)			0.001
Yes	39 (14.8)	58 (27.0)	
No	225 (85.2)	157 (73.0)	
SBP (mmHg)	137.80 ± 16.76	140.00 ± 18.97	0.178
DBP (mmHg)	79.58 ± 11.10	77.67 ± 11.62	0.068
Laboratory parameters			
HbA1c (%)	8.35 (7.03, 9.39)	9.21 (7.94, 10.75)	< 0.001
Serum albumin (g/L)	39.10 (37.40, 41.08)	38.00 (36.10, 39.20)	< 0.001
DBiL (μmol/L)	4.20 (3.33, 5.38)	4.10 (3.20, 5.30)	0.338
TBiL (μmol/L)	11.50 (8.83, 15.38)	10.90 (7.60, 13.60)	0.007
ALT (U/L)	19.50 (14.70, 30.70)	18.50 (13.20, 26.10)	0.015
AST (U/L)	16.60 (13.50, 22.58)	16.30 (13.40, 20.40)	0.177
SUN (mmol/L)	5.50 (4.50, 6.60)	5.90 (5.00, 8.00)	< 0.001
Scr (μmol/L)	66.00 (57.00, 74.00)	74.00 (65.00, 92.00)	< 0.001
eGFR (ml/min/1.73m^2^)	93.92 (84.29, 101.72)	87.87 (70.16, 96.16)	< 0.001
SUA (μmol/L)	318.00 (271.25, 375.75)	318.00 (271.00, 375.00)	0.794
FBG (mmol/L)	6.28 (5.17, 8.14)	7.17 (5.41, 9.81)	0.001
TC (mmol/L)	4.56 (3.77, 5.26)	4.19 (3.42, 5.08)	0.004
TG (mmol/L)	1.33 (0.91, 2.02)	1.32 (0.78, 1.83)	0.095
HDL-C (mmol/L)	1.02 (0.90, 1.21)	1.01 (0.90, 1.15)	0.445
LDL-C (mmol/L)	2.88 (2.08, 3.44)	2.39 (1.80, 3.11)	0.001
D-dimer (mg/L)	0.22 (0.20, 0.31)	0.25 (0.22, 0.43)	< 0.001
CRP (mg/L)	0.50 (0.10, 0.62)	0.50 (0.12, 0.64)	0.351
Neutrophils count (10^9^/L)	3.35 (2.60, 4.02)	3.36 (2.55, 4.19)	0.665
Lymphocyte count (10^9^/L)	2.05 (1.67, 2.49)	1.80 (1.45, 2.18)	< 0.001
NLR	1.56 (1.23, 2.04)	1.81 (1.37, 2.34)	< 0.001
Platelet count (10^9^/L)	210.00 (183.25, 249.00)	204.00 (176.00, 239.00)	0.077
25-OH VitD (ng/mL)	18.30 (14.63, 24.26)	18.30 (13.45, 23.75)	0.197
NSE (ng/mL)	14.90 (13.33, 16.50)	14.90 (13.10, 17.40)	0.765
Fasting insulin (mU/L)	6.10 (3.87, 11.96)	4.96 (2.19, 8.55)	< 0.001
Fasting C-peptide (ng/mL)	1.74 (1.32, 2.56)	1.68 (1.09, 2.38)	0.023
Ferritin (ng/mL)	210.00 (138.00, 294.50)	210.00 (163.00, 260.00)	0.367
Urinary microalbumin (mg/L)	11.90 (5.93, 16.00)	14.30 (8.00, 66.00)	< 0.001
Urinary creatinine (mmol/L)	7.24 (5.75, 11.07)	6.55 (4.33, 8.73)	< 0.001
UACR (mg/g)	12.26 (6.55, 16.05)	21.99 (9.78, 92.12)	< 0.001
Carotid stenosis			< 0.001
No stenosis	252 (95.5)	166 (87.3)	
< 50%	12 (4.5)	44 (11.7)	
≥ 50%	0 (0.0)	5 (1.0)	

*DPN* diabetic peripheral neuropathy, *BMI* body mass index, *T1DM* type 1 diabetes, *T2DM* type 2 diabetes, *SBP* systolic blood pressure, *DBP* diastolic blood pressure, *HbA1c* glycosylated hemoglobin, *DBiL* direct bilirubin, *TBiL* total bilirubin, *ALT* alanine aminotransferase, *AST* aspartate aminotransferase, *SUN* serum urea nitrogen, *Scr* serum creatinine, *eGFR* estimated glomerular filtration rate, *SUA* serum uric acid, *FBG* fasting blood glucose, *TC* total cholesterol, *TG* triglyceride, *HDL-C* high-density lipoprotein cholesterol, *LDL-C* low-density lipoprotein cholesterol, *CRP* C-reactive protein, *NLR* neutrophil-to-lymphocyte ratio, *NSE* neuron-specific enolase, *UACR* urinary albumin-to-creatinine ratio

**Table 2:** Clinical features in diabetic patients with or without LEAD

Clinical features	Non-LEAD (*n* = 207)	LEAD (*n* = 69)	*P* value
Demographic profiles			
Age (years)	63.00 (56.00, 69.00)	69.00 (65.00, 76.00)	< 0.001
Sex, n (%)			< 0.001
Male	107 (51.7)	55 (79.7)	
Female	100 (48.3)	14 (20.3)	
BMI (kg/m^2^)	24.33 (22.31, 27.00)	23.90 (21.75, 26.06)	0.123
Diabetes duration (year)	11.00 (6.00, 19.00)	15.00 (5.75, 20.00)	0.481
Type			0.207
T1DM	8 (3.9)	0 (0.0)	
T2DM	199 (96.1)	69 (100.00)	
Smoking history, n (%)			0.094
Yes	35 (16.9)	18 (26.1)	
No	172 (83.1)	51 (73.9)	
SBP (mmHg)	139.58 ± 17.10	141.48 ± 19.49	0.443
DBP (mmHg)	79.19 ± 10.97	76.55 ± 11.92	0.092
Laboratory parameters			
HbA1c (%)	8.86 (7.24, 9.61)	8.97 (8.01, 10.64)	0.018
Serum albumin (g/L)	38.50 (36.80, 40.20)	37.4 (34.90, 39.25)	0.002
DBiL (μmol/L)	4.10 (3.20, 5.10)	4.10 (3.35, 5.40)	0.697
TBiL (μmol/L)	10.90 (8.00, 14.40)	10.6 (7.9, 12.85)	0.297
ALT (U/L)	19.20 (13.70, 29.10)	19.40 (13.00, 30.1)	0.867
AST (U/L)	15.90 (13.50, 19.90)	16.70 (13.55, 24.90)	0.324
SUN (mmol/L)	5.60 (4.70, 6.70)	6.10 (5.00, 8.15)	0.009
Scr (μmol/L)	67.00 (59.00, 78.00)	76.00 (67.00, 94.00)	< 0.001
eGFR (ml/min/1.73m^2^)	93.58 (84.75, 101.74)	87.47 (64.76, 95.10)	< 0.001
SUA (μmol/L)	318.00 (280.00, 367.00)	319.00 (271.50, 371.5)	0.834
FBG (mmol/L)	6.50 (5.20, 8.45)	7.31 (5.47, 10.80)	0.022
TC (mmol/L)	4.43 (3.65, 5.16)	3.77 (3.23, 4.46)	0.001
TG (mmol/L)	1.33 (0.86, 1.98)	1.25 (0.79, 1.66)	0.335
HDL-C (mmol/L)	1.01 (0.91, 1.21)	0.98 (0.86, 1.06)	0.009
LDL-C (mmol/L)	2.68 (1.95, 3.37)	2.18 (1.68, 2.72)	0.007
D-dimer (mg/L)	0.23 (0.22, 0.30)	0.32 (0.23, 0.54)	< 0.001
CRP (mg/L)	0.50 (0.33, 0.62)	0.50 (0.35, 0.64)	0.405
Neutrophils count (10^9^/L)	3.35 (2.60, 4.15)	3.54 (2.87, 4.31)	0.183
Lymphocyte count (10^9^/L)	1.96 (1.58, 2.45)	1.79 (1.43, 2.09)	0.002
NLR	1.63 (1.30, 2.08)	2.11 (1.46, 2.66)	0.001
Platelet count (10^9^/L)	211.00 (187.00, 253.00)	197.00 (171.50, 244.00)	0.097
25-OH VitD (ng/mL)	18.30 (14.19, 24.66)	18.30 (12.51, 23.51)	0.505
NSE (ng/mL)	14.90 (13.30, 17.40)	14.80 (12.85, 17.70)	0.466
Fasting insulin (mU/L)	5.63 (2.87, 10.23)	5.31 (2.98, 7.94)	0.322
Fasting C-peptide (ng/mL)	1.72 (1.16, 2.49)	1.72 (1.20, 2.47)	0.782
Ferritin (ng/mL)	210.00 (153.00, 272.00)	210.00 (152.00, 277.5)	0.915
Urinary microalbumin (mg/L)	6.00 (13.00, 20.00)	21.00 (10.00, 104.50)	0.001
Urinary creatinine (mmol/L)	5.47 (3.94, 7.42)	5.28 (3.74, 6.45)	0.245
UACR (mg/g)	15.27 (7.67, 26.29)	23.04 (12.04, 166.76)	< 0.001
Carotid stenosis			< 0.001
No stenosis	194 (93.7)	44 (63.8)	
< 50%	9 (4.3)	24 (34.8)	
≥ 50%	4 (1.9)	1 (1.4)	

*LEAD* lower extremity arterial disease, *BMI* body mass index, *T1DM* type 1 diabetes, *T2DM* type 2 diabetes, *SBP* systolic blood pressure, *DBP* diastolic blood pressure, *HbA1c* glycosylated hemoglobin, *DBiL* direct bilirubin, *TBiL* total bilirubin, *ALT* alanine aminotransferase, *AST* aspartate aminotransferase, *SUN* serum urea nitrogen, *Scr* serum creatinine, *eGFR* estimated glomerular filtration rate, *SUA* serum uric acid, *FBG* fasting blood glucose, *TC* total cholesterol, *TG* triglyceride, *HDL-C* high-density lipoprotein cholesterol, *LDL-C* low-density lipoprotein cholesterol, *CRP* C-reactive protein, *NLR* neutrophil-to-lymphocyte ratio, *NSE* neuron-specific enolase, *UACR* urinary albumin-to-creatinine ratio

### Selected features

For DPN, a consensus was reached on eight features selected by all three feature selection methods. An additional four features were agreed upon by two of the methods, resulting in a total of 12 distinct features that were incorporated into the models, as outlined in Supplementary Table S3. Similarly, for LEAD, unanimous selection was achieved for five features across all methods, with another four features chosen by two of the methods. Thus, a total of nine features were integrated into the ML models, as detailed in Supplementary Table S4.

### Diagnostic performance of LR, RF, and XGBoost in detecting DPN

**Fig. 3:**
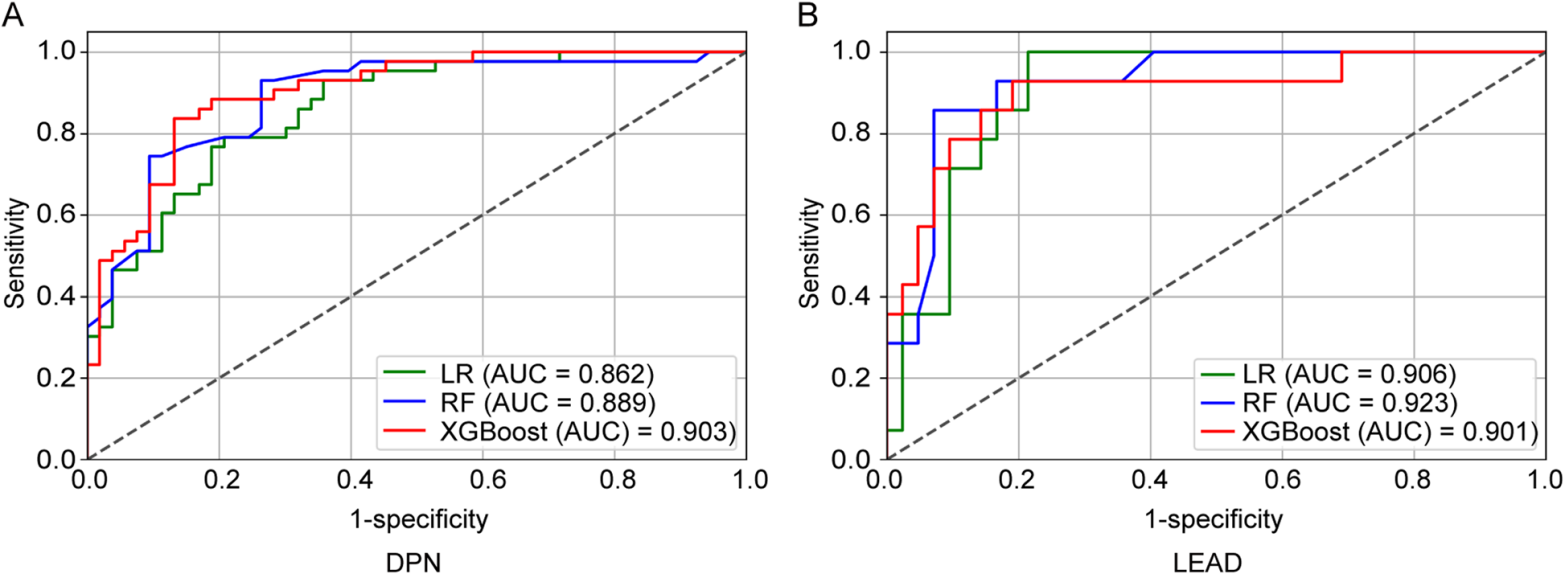
ROC curves of LR, RF, and XGBoost models for detecting DPN and LEAD. **A** ROC curves for DPN; **B** ROC curves for LEAD. ROC, receiver operating characteristic. LR, logistic regression. RF, random forest. DPN, diabetic peripheral neuropathy. LEAD, lower extremity arterial disease

**Fig. 4:**
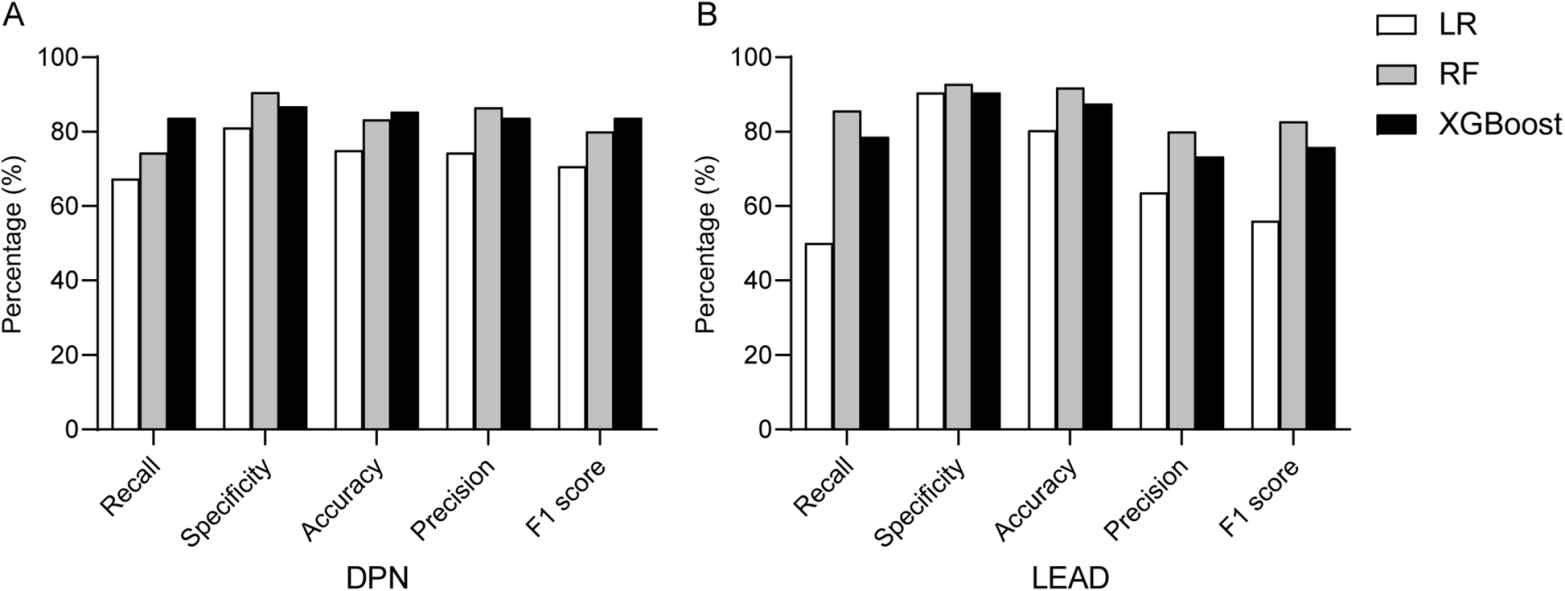
Performance of LR, RF, and XGBoost for detecting DPN and LEAD. **A** Performance for DPN models; **B** Performance for LEAD models. LR, logistic regression. RF, random forest. DPN, diabetic peripheral neuropathy. LEAD, lower extremity arterial disease

The diagnostic performances of three models for detecting DPN were shown in Figs. 3A and 4A. Among these models, XGBoost demonstrated the highest diagnostic efficacy, achieving an AUC of 0.903, a recall of 83.7%, a specificity of 86.8%, an accuracy of 85.4%, a precision of 83.7%, and an F1 score of 83.7%. Additionally, RF showed the highest specificity, at 90.6%.

### Diagnostic performance of LR, RF, and XGBoost in detecting LEAD

The performances of the LEAD models were presented in Figs. 3B and 4B. The RF model outperformed the others with the highest AUC of 0.923, recall of 85.7%, specificity of 92.9%, accuracy of 91.9%, precision of 80.0%, and an F1 score of 82.8%, followed by XGBoost and LR.

### Critical shared and unique risk factors for DPN and LEAD through SHAP analysis

**Fig. 5:**
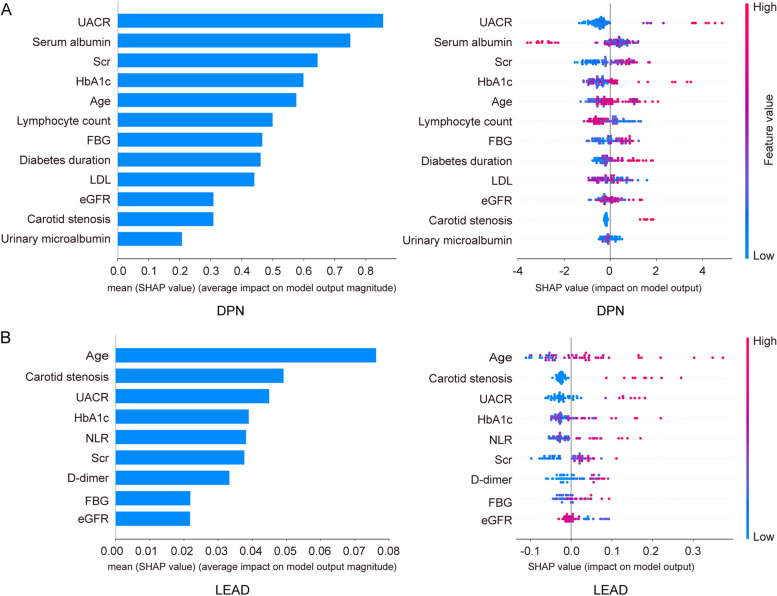
Feature importance of SHAP values for XGBoost model in detecting DPN and for RF model in detecting LEAD. **A** SHAP values of XGBoost model in detecting DPN; **B** SHAP values of RF model in detecting LEAD. SHAP, SHapley Additive exPlanation. RF, random forest. DPN, diabetic peripheral neuropathy. LEAD, lower extremity arterial disease

SHAP was applied to evaluate the importance of features within the optimal ML models for DPN and LEAD, with a prioritized list vividly illustrating their respective impacts. Figure 5A and B presented the rankings of critical features in the XGBoost model for DPN and the RF model for LEAD, respectively. This analysis highlighted the importance of both shared and unique risk factors. Common risk factors identified for both conditions include increased UACR, elevated HbA1c, elevated Scr, advanced age, carotid stenosis, high FBG, and reduced eGFR. Unique to DPN were decreased serum albumin and lower lymphocyte count, whereas LEAD was specifically associated with increased NLR and higher D-dimer levels.

## Discussion

This study constructed three different ML models for predicting DPN and LEAD among diabetic patients, utilizing basic clinical and laboratory data. We discovered that the XGBoost model demonstrated superior diagnostic performance in detecting DPN, whereas the RF model excelled in identifying LEAD. Furthermore, SHAP analysis identified the top five important risk factors common to both conditions: elevated UACR, HbA1c, Scr, advanced age, and carotid stenosis. Additionally, it pinpointed unique risk factors for each condition: a decrease in serum albumin and lymphocyte count were significant for DPN, while increased NLR and D-dimer were key indicators for LEAD.

ML models are significantly advancing the field of medical diagnostics. Recent advancements in predicting DPN and LEAD were summarized Table 3. For DPN detection, Metsker et al. [25] developed ML models using age, gender, and 27 laboratory tests. Among these models, the artificial neural network (ANN) achieved the highest recall at 0.809, the LR had the highest precision at 0.683, while Linear Regression displayed both the highest F1 score at 0.730 and the highest accuracy at 0.747. Another study demonstrated that, using demographic, clinical, and laboratory data, both RF and SVM models significantly distinguished DPN in individuals with T2DM. The accuracy, sensitivity, and specificity were 67.8%, 68.09%, and 67.44% for RF, and 67.8%, 68.89%, and 66.67% for SVM, respectively [26]. By contrast, our study showed that the XGBoost model had the highest diagnostic performance, with an accuracy of 85.4%, a sensitivity of 83.7%, and a specificity of 86.8%, which were much higher than those reported in previous research. For LEAD, our RF model showed superior performance, aligning with previous findings that highlighted the RF model’s enhanced predictive capabilities over the LR model [4]. Of note, the improved performance in previous studies was attributed to the inclusion of the ankle-brachial pressure index (ABI), a common indicator for diagnosing LEAD. Our study, however, relied solely on clinical data and routine laboratory tests to construct ML models. The remarkable performance of our models can be attributed to our methods of feature selection and hyperparameter optimization. We combined three different methods—MI, RF-RFE, and the Boruta algorithm—to identify the most significant features. This approach significantly reduces overfitting and enhances the robustness of our models. Besides, PSO was applied to optimize hyperparameters. Unlike traditional methods such as grid search, PSO does not rely on fixed parameter value range and step size, making it particularly effective for complex optimization challenges with large parameter spaces.

**Table 3:** Comparative analysis of the proposed work with previous studies for DPN and LEAD prediction models

Studies	Features	Models	Results
DPN			
Lian et al. [11]	medical records and laboratory tests	LR, k-nearest neighbor, decision tree, naive bayes, RF, and XGBoost	The XGBoost model outperformed others with an accuracy of 0.746, precision of 0.765, recall of 0.711, F1-score of 0.736, and AUC of 0.813
Metsker et al. [25]	age, gender, 27 laboratory tests	ANN, SVM, Decision tree, Liner Regression, LR	The highest recall (0.809) for ANN, precision (0.683) for LR, and F1 score (0.730) and accuracy (0.747) for Liner Regression
Rashid et al. [26]	demographic, clinical, and laboratory profiles	LR, RF, and SVM	Both RF and SVM models reached a peak accuracy of 67.8%, with RF showing 68.09% recall and 67.44% specificity, and SVM at 68.89% recall and 66.67% specificity
Proposed work	demographic, clinical, and laboratory data	RF, LR, XGBoost	XGBoost showing the highest performance, with an accuracy of 85.4%, a recall of 83.7%, a specificity of 86.8%, a precision of 83.7%, and an AUC of 0.903
LEAD			
Gao et al. [4]	clinical and laboratory features	RF, LR	RF showing a sensitivity of 90.7%, a specificity of 90.4%, and an accuracy of 91.2%
Proposed work	demographic, clinical, and laboratory data	RF, LR, XGBoost	RF excelled, with an accuracy of 91.9%, a recall of 85.7%, a specificity of 92.9%, a precision of 80.0%, and an AUC of 0.923

*DPN* diabetic peripheral neuropathy, *LEAD* lower extremity arterial disease, *LR* logistic regression, *ANN* artificial neural network, *SVM* support vector machine, *RF* random forest

SHAP, a game theory-based method, was used in this study to identify key risk factors for DPN and LEAD. The analysis revealed that the primary risk factors common to both conditions were increased UACR, HbA1c, Scr, advanced age, and carotid stenosis. Notably, UACR was ranked as the most crucial predictor for DPN and the third most significant for LEAD. This finding was consistent with a large retrospective cohort study that identified UACR as a crucial predictor for DPN [27]. Additionally, a 30% or greater increase in UACR was reported to be a risk factor for the onset of DPN [28]. Previous studies also discovered that UACR served as a biomarker for the early detection of LEAD [29] and a risk factor for mortality in LEAD patients [30]. This underscored the critical importance of regular UACR monitoring to prevent DPN and LEAD, thereby potentially reducing the risk of DFUs.

As expected, HbA1c and older age were critical shared risk factors for both conditions, aligning with previous studies [27, 31–33]. Unlike FBG, which can fluctuate significantly due to various factors, HbA1c provides a more stable measure of blood glucose levels over the preceding three months. Chronic hyperglycemia in diabetes contributed to the development of DPN through mechanisms such as increased oxidative stress and inflammation [34]. These processes disrupt blood flow to peripheral nerves and impair nerve function. Chronic hyperglycemia can also cause damage to endothelial cells and thicken the intima-media layer in blood vessels, particularly in the lower extremities [35]. Additionally, as individuals age, the key components of the extracellular matrix, particularly elastic fibers, are subjected to degradation and fragmentation. Age-related increases in cross-linking between collagen fibers could further contribute to the development of arterial stiffness [36], which may diminish blood flow to nerves and affect their repair capabilities, potentially increasing the prevalence of DPN [37]. These findings underscored the importance of maintaining good glycemic control, especially in older patients.

Scr is a key marker for kidney function, with elevated levels often indicating renal damage. Impaired kidney function can affect the microcirculation in distant organs [38], potentially compromising blood flow to peripheral nerves and arteries, which increases the risk of DPN and LEAD. Moreover, carotid stenosis was also recognized as a significant risk factor for both conditions. While the direct link between carotid stenosis and DPN is less studied, recent findings suggested that carotid atherosclerosis, the primary cause of carotid stenosis, could independently predict small fiber nerve dysfunction in individuals with T2DM [39]. Furthermore, a cross-sectional study of 653 patients with LEAD found that 415 (63.5%) had carotid stenosis [40], implying that carotid stenosis may be a contributing risk factor for LEAD. Therefore, diabetic patients should also pay more attention on kidney function and neck vascular health to reduce the prevalence of DPN and LEAD.

Furthermore, unique risk factors were also identified. For DPN, decreased serum albumin was a critical predictor. Among patients with T2DM, a serum albumin level below 36.75g/L was independently associated with impaired peripheral nerve function, with a sensitivity of 65.6% and a specificity of 78.0% for detecting abnormal function in those with albuminuria [41]. Recent studies further supported the inverse relationship between serum albumin levels and the prevalence of DPN among T2DM patients [42, 43]. These findings suggest that serum albumin may play a protective role against the development of DPN, potentially due to its antioxidant, anti-inflammatory, and anti-atherosclerotic properties [42]. Another unique but often overlooked risk factor for DPN was lymphocyte count. Both serum albumin and lymphocyte count are indicators for nutritional status [44], highlighting the importance for patients with DPN to closely monitor and manage their nutrition.

For LEAD, elevated NLR was identified as a unique key risk factor, consistent with previous studies [45, 46], which discovered that NLR was positively related with the prevalence of LEAD. D-dimer was identified as another crucial predictor for LEAD. In a prospective cohort study, patients with LEAD had significant higher levels of D-dimer than those without LEAD [47]. In addition, the levels of D-dimer were observed to increase with the severity of LEAD [48]. Elevated D-dimer levels may reflect the extent of atherosclerosis, as they indicate ongoing fibrin formation and degradation [49].

## Conclusion

Our study underscored the potential of ML models in predicting DPN and LEAD among diabetic patients. We found that XGBoost showed superior performance in identifying DPN, whereas RF model was more effective for diagnosing LEAD. SHAP analysis revealed the top five most critical risk factors common to both conditions, including elevated UACR, HbA1c, Scr, advanced age, and carotid stenosis. Additionally, unique predictors were identified for each condition: decreased serum albumin and lymphocyte count were associated with DPN, whereas increased NLR and D-dimer levels were linked to LEAD. These insights underscored the complexity of managing DPN and LEAD, emphasizing the need for personalized and comprehensive treatment strategies. Implementing these insights could enhance early detection and management of these diabetic complications, particularly beneficial in regions with limited medical resources. Prioritizing the management of shared risk factors, like glycemic control, renal function, and macrovascular health, may reduce the frequency of DPN and LEAD, thereby decreasing the risk of DFUs. Patients with DPN should also focus on maintaining good nutritional health. For future progress, research should be expanded to include a broader and more diverse population, and investigate the feasibility of developing a unified ML model capable of predicting both DPN and LEAD in individuals with diabetes.

### Supplementary Information

Supplementary Material 1.

Supplementary Material 2.

## Data Availability

Some or all datasets generated during and analyzed during the current study are not publicly available but are available from the corresponding author on reasonable request.

## Abbreviations

DPN: Diabetic peripheral neuropathy
LEAD: Lower extremity arterial disease
DFUs: Diabetic foot ulcers
T2DM: Type 2 diabetes mellitus
MI: Mutual information
RF-RFE: Random forest recursive feature elimination
ML: Machine learning
LR: Logistic regression
RF: Random forest
XGBoost: EXtreme Gradient Boosting
SVM: Support vector machine
PSO: Particle swarm optimization
SHAP: SHapley Additive exPlanation
ROC: Receiver operating characteristic
AUC: Area under the receiver operating curve
UACR: Urinary albumin-to-creatinine ratio
EMG: Electromyography
DKA: Diabetic ketoacidosis
HHS: Hyperosmotic hyperglycemia syndrome
BMI: Body mass index
SBP: Systolic blood pressure
DBP: Diastolic blood pressure
NLR: Neutrophil-to-lymphocyte ratio
ALT: Alanine aminotransferase
AST: Aspartate aminotransferase
TBiL: Total bilirubin
DBiL: Direct bilirubin
SUN: Serum urea nitrogen
SUA: Serum uric acid
Scr: Serum creatinine
FBG: Fasting blood glucose
HbA1c: Glycosylated hemoglobin
TC: Total cholesterol
HDL: High-density lipoprotein
LDL: Low-density lipoprotein
TG: Triglycerides
CRP: C-reactive protein
25-OH VitD: 25-Hydroxy vitamin D3
NSE: Neuron specific enolase
eGFR: Estimated glomerular filtration rate
