# Supplementary material for: Interpretable machine learning models for detecting peripheral neuropathy and lower extremity arterial disease in diabetics: an analysis of critical shared and unique risk factors
Source: BMC Med Inform Decis Mak. 2024 Jul 22;24:200. doi: 10.1186/s12911-024-02595-z (PMC11265186; doi:10.1186/s12911-024-02595-z)
Supplement: Supplementary file 1 — Supplementary Material 1. [file 12911_2024_2595_MOESM1_ESM.docx]

**Supplementary file 1:**

**Supplementary Table S1.** Clinical features in diabetic patients; **Supplementary Table S2.** Optimal hyperparameter values for ML models using PSO; **Supplementary Table S3**. Selecting the most important features for DPN; **Supplementary Table S4**. Selecting the most important features for LEAD.

Table S1 Clinical features in diabetic patients.

| Clinical features | Description | Type | n=479 |
| --- | --- | --- | --- |
| Demographic profiles |  |  |  |
| Age | years | numerical | 65.00 (59.00, 70.00) |
| Sex | Male=1, Female=0 | categorical | 1/2 (266/213) |
| BMI | kg/m^2^ | numerical | 24.33 (22.22, 26.82) |
| Diabetes duration | year | numerical | 12.00 (5.50, 19.00) |
| Type | T1DM=1, T2DM=2 | categorical | 1/2 (11/468) |
| Smoking history, n (%) | Yes=1, No=0 | categorical | 1/0 (382/97) |
| SBP | mmHg | numerical | 138.00 (127.00, 151.00) |
| DBP | mmHg | numerical | 78.72±11.36 |
| Laboratory parameters |  |  |  |
| HbA1c | % | numerical | 8.95 (7.27, 9.90) |
| Serum albumin | g/L | numerical | 38.50 (36.70, 39.90) |
| DBiL | μmol/L | numerical | 4.10 (3.20, 5.30) |
| TBiL | μmol/L | numerical | 11.30 (8.30, 14.40) |
| ALT | U/L | numerical | 19.20 (13.70, 29.10) |
| AST | U/L | numerical | 16.50 (13.40, 21.60) |
| SUN | mmol/L | numerical | 5.60 (4.70, 7.10) |
| Scr | μmol/L | numerical | 69.00 (60.00, 83.00) |
| eGFR | ml/min/1.73m^2^ | numerical | 91.88 (78.03, 99.01) |
| SUA | μmol/L | numerical | 318.00 (271.00, 375.00) |
| FBG | mmol/L | numerical | 6.69 (5.26, 8.72) |
| TC | mmol/L | numerical | 4.43 (3.61, 5.18) |
| TG | mmol/L | numerical | 1.33 (0.87, 1.95) |
| HDL-C | mmol/L | numerical | 1.01 (0.90, 1.18) |
| LDL-C | mmol/L | numerical | 2.68 (1.96, 3.36) |
| D-dimer | mg/L | numerical | 0.24 (0.22, 0.35) |
| CRP | mg/L | numerical | 0.50 (0.50, 0.50) |
| Neutrophils count | 10^9^/L | numerical | 3.35 (2.56, 4.09) |
| Lymphocyte count | 10^9^/L | numerical | 1.92 (1.56, 2.37) |
| NLR |  | numerical | 1.67 (1.28, 2.15) |
| Platelet count | 10^9^/L | numerical | 209.00 (181.00, 245.00) |
| 25-OH VitD | ng/mL | numerical | 18.30 (14.19, 24.08) |
| NSE | ng/mL | numerical | 14.90 (13.20, 17.00) |
| Fasting insulin | mU/L | numerical | 5.63 (2.94, 10.22) |
| Fasting C-peptide | ng/mL | numerical | 1.72 (1.17, 2.49) |
| Ferritin | ng/mL | numerical | 210.00 (151.00, 272.00) |
| Urinary microalbumin | mg/L | numerical | 14.00 (6.60, 26.10) |
| Urinary creatinine | mmol/L | numerical | 7.24 (4.91, 9.88) |
| UACR | mg/g | numerical | 15.97 (7.68, 30.47) |
| Carotid stenosis | No=0, <50%=1, ≥50%=2 | categorical | 0/1/2 (418/56/5) |

DPN, diabetic peripheral neuropathy. BMI, body mass index. T1DM, type 1 diabetes. T2DM, type 2 diabetes. SBP, systolic blood pressure. DBP, diastolic blood pressure. HbA1c, glycosylated hemoglobin. DBiL, direct bilirubin. TBiL, total bilirubin. ALT, alanine aminotransferase. AST, aspartate aminotransferase. SUN, serum urea nitrogen. Scr, serum creatinine. eGFR, estimated glomerular filtration rate. SUA, serum uric acid. FBG, fasting blood glucose. TC, total cholesterol. TG, triglyceride. HDL-C, high-density lipoprotein cholesterol. LDL-C, low-density lipoprotein cholesterol. CRP, C-reactive protein. NLR, neutrophil-to-lymphocyte ratio. NSE, neuron-specific enolase. UACR, urinary albumin-to-creatinine ratio.

Table S2 Optimal hyperparameter values for ML models using PSO.

| Models | Hyperparameter tuning | Optimal value (DPN) | Optimal value (LEAD) |
| --- | --- | --- | --- |
| LR | 'C': [0.01, 200],  'solver': ['newton-cg', 'lbfgs', 'liblinear', 'sag', 'saga'] | C=102.34,  solver=’ lbfgs’,  penalty='l2' (default) | C=194.76,  solver= ‘liblinear’,  penalty='l2' (default) |
| RF | 'n_estimators': [10, 200],  'max_depth': [1, 50],  'random_state': [1, 100] | n_estimators=74, max_depth=23, random_state=83 | n_estimators=110, max_depth=27, random_state=17 |
| XGBoost | 'learning_rate': [0.01,0.3],  'max_depth': [3, 10],  'subsample': [0.5,1] | learning_rate=0.18,  max_depth=8,  subsample=0.93 | learning_rate= 0.17,  max_depth=9,  subsample=0.99 |

ML, machine learning. PSO, particle swarm optimization. DPN, diabetic peripheral neuropathy. LEAD, lower extremity arterial disease. LR, logistic regression. RF, random forest.

Table S3 Selecting the most important features for DPN.

| Features | MI | RF-RFE | Boruta | Score |
| --- | --- | --- | --- | --- |
| Age | √ | √ | √ | 3 |
| Gender | √ |  |  | 1 |
| Diabetes duration | √ | √ | √ | 3 |
| BMI |  | √ |  | 1 |
| HbA1c | √ | √ | √ | 3 |
| AST | √ |  |  | 1 |
| Albumin | √ | √ | √ | 3 |
| SUN |  |  | √ | 1 |
| Scr |  | √ | √ | 2 |
| eGFR | √ | √ | √ | 3 |
| FBG |  | √ | √ | 2 |
| Lymphocyte count |  | √ | √ | 2 |
| CRP | √ |  |  | 1 |
| Urinary microalbumin | √ | √ | √ | 3 |
| Urinary creatinine |  | √ |  | 1 |
| UACR | √ | √ | √ | 3 |
| HDL-C | √ |  |  | 1 |
| LDL-C | √ | √ | √ | 3 |
| Fasting insulin |  | √ |  | 1 |
| Fasting C-peptide | √ |  |  | 1 |
| 25-OH VitD | √ |  |  | 1 |
| Carotid stenosis | √ | √ |  | 2 |

DPN, diabetic peripheral neuropathy. MI, mutual information. RF-REF, random forest recursive feature elimination. BMI, body mass index. HbA1c, glycosylated hemoglobin. AST, aspartate aminotransferase. SUN, serum urea nitrogen. Scr, serum creatinine. eGFR, estimated glomerular filtration rate. FBG, fasting blood glucose. CRP, C-reactive protein. UACR, urinary albumin-to-creatinine ratio. HDL-C, high-density lipoprotein cholesterol. LDL-C, low-density lipoprotein cholesterol.

Table S4 Selecting the most important features for LEAD.

| Features | MI | RF-RFE | Boruta | Score |
| --- | --- | --- | --- | --- |
| Age | √ | √ | √ | 3 |
| Type | √ |  |  | 1 |
| Diabetes duration | √ |  |  | 1 |
| DBP |  | √ |  | 1 |
| HbA1c |  | √ | √ | 2 |
| FBG |  | √ | √ | 2 |
| AST | √ |  |  | 1 |
| BUN | √ |  |  | 1 |
| Scr | √ | √ | √ | 3 |
| eGFR | √ | √ |  | 2 |
| SUA | √ |  |  | 1 |
| TC |  | √ |  | 1 |
| TG | √ |  |  | 1 |
| HDL-C |  | √ |  | 1 |
| D-dimer | √ | √ | √ | 3 |
| Neutrophils count | √ |  |  | 1 |
| Lymphocyte count |  | √ |  | 1 |
| NLR |  | √ | √ | 2 |
| Platelet count |  | √ |  | 1 |
| 25-OH VitD | √ |  |  | 1 |
| NSE | √ |  |  | 1 |
| Urinary microalbumin |  | √ |  | 1 |
| UACR | √ | √ | √ | 3 |
| Carotid stenosis | √ | √ | √ | 3 |

LEAD, lower extremity arterial disease. MI, mutual information. RF-REF, random forest recursive feature elimination. DBP, diastolic blood pressure. HbA1c, glycosylated hemoglobin. FBG, fasting blood glucose. AST, aspartate aminotransferase. SUN, serum urea nitrogen. TC, total cholesterol. TG, triglyceride. HDL-C, high-density lipoprotein cholesterol. NLR, neutrophil-to-lymphocyte ratio. NSE, neuron-specific enolase. UACR, urinary albumin-to-creatinine ratio.
