# Supplementary material for: Interpretable machine learning models for detecting peripheral neuropathy and lower extremity arterial disease in diabetics: an analysis of critical shared and unique risk factors
Source: BMC Med Inform Decis Mak. 2024 Jul 22;24:200. doi: 10.1186/s12911-024-02595-z (PMC11265186; doi:10.1186/s12911-024-02595-z)
Supplement: Supplementary file 2 — Supplementary Material 2. [file 12911_2024_2595_MOESM2_ESM.docx]

**Supplementary file 2:**

**The pseudo-code of ML models construction and interpretation**

import pandas as pd

from sklearn.ensemble import RandomForestClassifier

from sklearn.feature_selection import SelectKBest, mutual_info_classif, RFE

from boruta import BorutaPy

import numpy as np

from sklearn.model_selection import train_test_split

**# 1. Feature selection**

**# Load the dataset**

filename = 'DPN_dataset.xlsx'

df = pd.read_excel(filename)

X = df.iloc[:, :-1] # Assume that the features are in all columns except the last

y = df.iloc[:, -1] # Assume that the target variable is in the last column

**# Split into training and test sets**

from sklearn.model_selection import train_test_split

X_train, X_test, y_train, y_test = train_test_split(X, y, test_size=0.2, random_state=30)

**# 1.1 Feature selection using mutual information (MI)**

from sklearn.feature_selection import SelectKBest, mutual_info_classif

# Select 15 best features using mutual information

selector = SelectKBest(mutual_info_classif, k=15)

# Retrieve the names of the selected features

MI_selected_features = X_train.columns[selector.get_support()]

print("MI_selected_features:", MI_selected_features)

**# 1.2 Feature selection using random forest recursive feature elimination (RF-RFE)**

# Create a RandomForest classifier

rfc = RandomForestClassifier(n_estimators=100, random_state=0)

# Perform feature selection using RF-RFE

rfe = RFE(estimator=rfc, n_features_to_select=10, step=1)

# Fit the RF-RFE model using only the training set

rfe.fit(X_train, y_train)

# Transform the data

X_train_RFE = rfe.transform(X_train)

# Select the important features

RFE_selected_features = df.columns[:-1][rfe.support_].tolist()

print("RFE_selected_features:", RFE_selected_features)

**# 1.3 Feature selection using the Boruta algorithm**

model = RandomForestClassifier(n_estimators=100, random_state=0)

feat_selector = BorutaPy(model, n_estimators='auto', verbose=2, random_state=1)

feat_selector.fit(np.array(X_train), np.array(y_train))

# Mark the confirmed features

confirmed_features = features[feat_selector.support_]

# Mark the tentative features

tentative_features = features[feat_selector.support_weak_]

# Mark the rejected features

rejected_features = features[~feat_selector.support_ & ~feat_selector.support_weak_]

# Combine confirmed and tentative features

Boruta_selected features = confirmed_features.tolist() + tentative_features.tolist()

print("Boruta_selected features:", Boruta_selected features)

**# 2. Model construction**

**# 2.1 Random forest model construction with particle swarm optimization (PSO) for fine-tuning hyperparameters**

import pandas as pd

from sklearn.ensemble import RandomForestClassifier

from sklearn.metrics import roc_auc_score, recall_score, confusion_matrix, accuracy_score, precision_score, f1_score, roc_curve, auc

from sklearn.model_selection import train_test_split

from pyswarm import pso

# Load the dataset

filename = 'DPN_dataset.xlsx'

df = pd.read_excel(filename)

X = df.iloc[:, :-1] # Features are in all columns except the last

y = df.iloc[:, -1] # Target variable is in the last column

# Split into training and test sets

X_train, X_test, y_train, y_test = train_test_split(X, y, test_size=0.2, random_state=30)

def objective_function(hyperparameters):

n_estimators, max_depth, random_state = int(hyperparameters[0]), int(hyperparameters[1]), int(hyperparameters[2])

model = RandomForestClassifier(n_estimators=n_estimators, max_depth=max_depth, random_state=random_state)

model.fit(X_train, y_train)

y_pred = model.predict(X_test)

y_pred_proba = model.predict_proba(X_test)[:, 1]

# Calculate metrics

auc_score = roc_auc_score(y_test, y_pred_proba)

recall = recall_score(y_test, y_pred)

tn, fp, fn, tp = confusion_matrix(y_test, y_pred).ravel()

specificity = tn / (tn + fp)

# Return the negative sum of the AUC, recall, and specificity scores to facilitate minimization

return -(auc_score + recall + specificity)

# Set bounds for PSO

lb = [10, 1, 1] # Lower bounds

ub = [200, 50, 100] # Upper bounds

# Run PSO

xopt, fopt = pso(objective_function, lb, ub, swarmsize=30, maxiter=1, debug=True)

# Output optimal hyperparameters and their combined score

print("Optimal number of estimators:", int(xopt[0]))

print("Optimal max depth:", int(xopt[1]))

print("Optimal random state:", int(xopt[2]))

print("Optimal combined score:", -(fopt))

# Initialize and fit the final model with optimal parameters

best_model_RF = RandomForestClassifier(n_estimators=int(xopt[0]), max_depth=int(xopt[1]), random_state=int(xopt[2]))

best_model_RF.fit(X_train, y_train)

# Evaluate the model

y_pred_rf = best_model_RF.predict(X_test)

y_pred_proba_rf = best_model_RF.predict_proba(X_test)[:,1]

fpr_rf, tpr_rf, thresholds = roc_curve(test_flag, y_pred_proba_rf)

conf_matrix_rf = confusion_matrix(y_test, y_pred_rf)

model_RF _auc=auc(fpr_rf,tpr_rf)

accuracy = accuracy_score(y_test, y_pred_rf)

recall = recall_score(y_test, y_pred_rf)

precision = precision_score(y_test, y_pred_rf)

f1 = f1_score(y_test, y_pred_rf)

specificity = conf_matrix_rf[0][0] / (conf_matrix_rf[0][0] + conf_matrix_rf[0][1])

# Print results

print("model_RF_auc=", model_RF_auc)

print(f"Accuracy: {accuracy}")

print(f"Recall: {recall}")

print(f"Precision: {precision}")

print(f"F1 Score: {f1}")

print(f"Specificity: {specificity}")

**# 2.2 XGBoost model construction with particle swarm optimization (PSO) for fine-tuning hyperparameters**

import numpy as np

import pandas as pd

from pyswarm import pso

from xgboost import XGBClassifier

from sklearn.model_selection import train_test_split

from sklearn.metrics import confusion_matrix, roc_auc_score,roc_curve, auc, accuracy_score, recall_score, precision_score, f1_score

# Load the dataset

filename = 'DPN_dataset.xlsx'

df = pd.read_excel(filename)

X = df.iloc[:, :-1] # Features are in all columns except the last

y = df.iloc[:, -1] # Target variable is in the last column

# Split into training and test sets

X_train, X_test, y_train, y_test = train_test_split(X, y, test_size=0.2, random_state=30)

def pso_optimize(params):

learning_rate, max_depth, subsample = params

max_depth = int(max_depth)

model = XGBClassifier(

learning_rate=learning_rate,

max_depth=max_depth,

subsample=subsample,

n_estimators=100,

use_label_encoder=False,

eval_metric='mlogloss'

)

model.fit(X_train, y_train)

y_pred = model.predict(X_test)

y_pred_proba = model.predict_proba(X_test)[:, 1]

# Calculate metrics

roc_auc = roc_auc_score(y_test, y_pred_proba)

recall = recall_score(y_test, y_pred)

tn, fp, fn, tp = confusion_matrix(y_test, y_pred).ravel()

specificity = tn / (tn + fp)

# Return negative sum to minimize

return -(roc_auc + recall + specificity)

# Set bounds for PSO

lb = [0.01, 3, 0.5] # Lower bounds

ub = [0.3, 10, 1.0] # Upper bounds

# Run PSO

best_params, best_score = pso(pso_optimize, lb, ub, swarmsize=50, maxiter=1)

print("Best Parameters:", best_params)

print("Best Combined Score:", -best_score)

# Extract best parameters

best_learning_rate, best_max_depth, best_subsample = best_params

best_max_depth = int(best_max_depth)

# Train final model

best_model_xgboost = XGBClassifier(

learning_rate=best_learning_rate,

max_depth=best_max_depth,

subsample=best_subsample,

n_estimators=100,

use_label_encoder=False,

eval_metric='mlogloss'

)

best_model_xgboost.fit(X_train, y_train)

# Evaluate the model

y_pred_xgboost = best_model_xgboost.predict(X_test)

y_pred_proba_xgboost = best_model_xgboost.predict_proba(X_test)[:, 1]

fpr_xgboost, tpr_xgboost, thresholds = roc_curve(y_test, y_pred_proba_xgboost)

accuracy = accuracy_score(y_test, y_pred_xgboost)

recall = recall_score(y_test, y_pred_xgboost)

precision = precision_score(y_test, y_pred_xgboost)

f1 = f1_score(y_test, y_pred_xgboost)

conf_matrix_xgboost = confusion_matrix(y_test, y_pred_xgboost)

specificity = conf_matrix_xgboost[0][0] / (conf_matrix_xgboost[0][0] + conf_matrix_xgboost[0][1])

model_xgboost_auc=auc(fpr_xgboost,tpr_xgboost)

# Print results

print("model_xgboost_auc=",model_xgboost_auc)

print(f"Accuracy: {accuracy}")

print(f"Recall: {recall}")

print(f"Precision: {precision}")

print(f"F1 Score: {f1}")

print(f"Specificity: {specificity}")

**# 2.3 Logistic model construction with particle swarm optimization (PSO) for fine-tuning hyperparameters**

import numpy as np

import pandas as pd

from pyswarm import pso

from sklearn.linear_model import LogisticRegression

from sklearn.model_selection import train_test_split

from sklearn.metrics import roc_auc_score,confusion_matrix, roc_curve, auc, accuracy_score, recall_score, precision_score, f1_score

# Load the dataset

filename = 'DPN_dataset.xlsx'

df = pd.read_excel(filename)

X = df.iloc[:, :-1] # Features are in all columns except the last

y = df.iloc[:, -1] # Target variable is in the last column

# Split into training and test sets

X_train, X_test, y_train, y_test = train_test_split(X, y, test_size=0.2, random_state=30)

def objective_function(params):

C = params[0]

solver_index = int(round(params[1]))

solver_index = min(max(solver_index, 0), len(solvers) - 1)

solver = solvers[solver_index]

model = LogisticRegression(C=C, max_iter=1000, penalty='l2', solver=solver)

model.fit(X_train, y_train)

y_pred = model.predict(X_test)

y_pred_proba = model.predict_proba(X_test)[:, 1]

# Calculate metrics

roc_auc = roc_auc_score(y_test, y_pred_proba)

recall = recall_score(y_test, y_pred)

tn, fp, fn, tp = confusion_matrix(y_test, y_pred).ravel()

specificity = tn / (tn + fp)

# Return negative sum to minimize

return -(roc_auc + recall + specificity)

# Set bounds for PSO

lb = [0.001, 0] # Lower bounds

ub = [20, len(solvers) - 1] # Upper bounds

# Run PSO

xopt, fopt = pso(objective_function, lb, ub, swarmsize=100, maxiter=2, debug=True)

# Extract best parameters

best_C = xopt[0]

best_solver = solvers[int(round(xopt[1]))]

print("Best C:", best_C)

print("Best Solver:", best_solver)

# Train final model

best_logistic_model = LogisticRegression(C=best_C, max_iter=100, penalty='l2', solver=best_solver)

best_logistic_model.fit(X_train, y_train)

# Evaluate the model

y_pred_LR = best_logistic_model.predict(X_test)

y_pred_proba_LR = best_logistic_model.predict_proba(X_test)[:,1]

fpr_LR, tpr_LR, thresholds = roc_curve(y_test,y_ y_pred_proba_LR)

accuracy = accuracy_score(y_test, y_pred_LR)

recall = recall_score(y_test, y_pred_LR)

precision = precision_score(y_test, y_pred_LR)

f1 = f1_score(y_test, y_pred_LR)

conf_matrix_LR = confusion_matrix(y_test, y_pred_LR)

specificity = conf_matrix_LR[0][0] / (conf_matrix_LR[0][0] + conf_matrix_LR[0][1])

model_LR_auc=auc(fpr_LR,tpr_LR)

# Print results

print("model_LR_auc=",model_LR_auc)

print(f"Accuracy: {accuracy}")

print(f"Recall: {recall}")

print(f"Precision: {precision}")

print(f"F1 Score: {f1}")

print(f"Specificity: {specificity}")

**# 3. SHAP interpretation**

import shap

import matplotlib.pyplot as plt

# Initialize JavaScript visualization in Jupyter Notebooks

shap.initjs()

# Assume `model_rf` is your pre-trained random forest model and `X_train` is your feature set

explainer_rf = shap.TreeExplainer(model_rf)

shap_values_rf = explainer_rf.shap_values(X_train)

# Creating an SHAP Explanation object for positive class predictions

explainer1 = shap.Explanation(values=shap_values_rf[1],

base_values=explainer_rf.expected_value[1], # Index depends on the class of interest

data=test_feature, # The feature data for model input

feature_names=test_feature.columns) # Feature names, assuming test_feature is a DataFrame

# Generate and display a beeswarm plot

fig, ax = plt.subplots()

shap.plots.beeswarm(explainer1, max_display=20, show=False)

# Save the beeswarm plot to a file

fig.savefig("shap_beeswarm_plot_rf.tif", dpi=300)

# Bar SHAP plot

# Select the SHAP values for the positive class

shap_values_positive = shap_values_rf[1]

# Create a SHAP summary bar plot

shap.summary_plot(shap_values_positive, test_feature, plot_type="bar")

# Save the bar plot image

plt.savefig('shap_summary_plot.tif', bbox_inches='tight')
